# Supplementary material for: B-Helper Neutrophils in Regional Lymph Nodes Correlate with Improved Prognosis in Patients with Head and Neck Cancer
Source: Cancers (Basel). 2021 Jun 21;13(12):3092. doi: 10.3390/cancers13123092 (PMC8234083; doi:10.3390/cancers13123092)
Supplement: Supplementary file 1 [file cancers-13-03092-s001.zip › cancers-1193551-supplementary.pdf]

cancers MDPI

Supplementary Files

B-helper Neutrophils in Regional Lymph Nodes Correlate with Improved Prognosis in Patients with Head and Neck Cancer

Ekaterina Pylaeva , Irem Ozel, Anthony Squire, Ilona Spyra, Charlotte Wallner, Magdalena Korek, Georg Korschunow, Maksim Domnich, Elena Siakaeva, Moritz Goetz, Agnes Bankfalvi, Stephan Lang, Benjamin Kansy and Jadwiga Jablonska

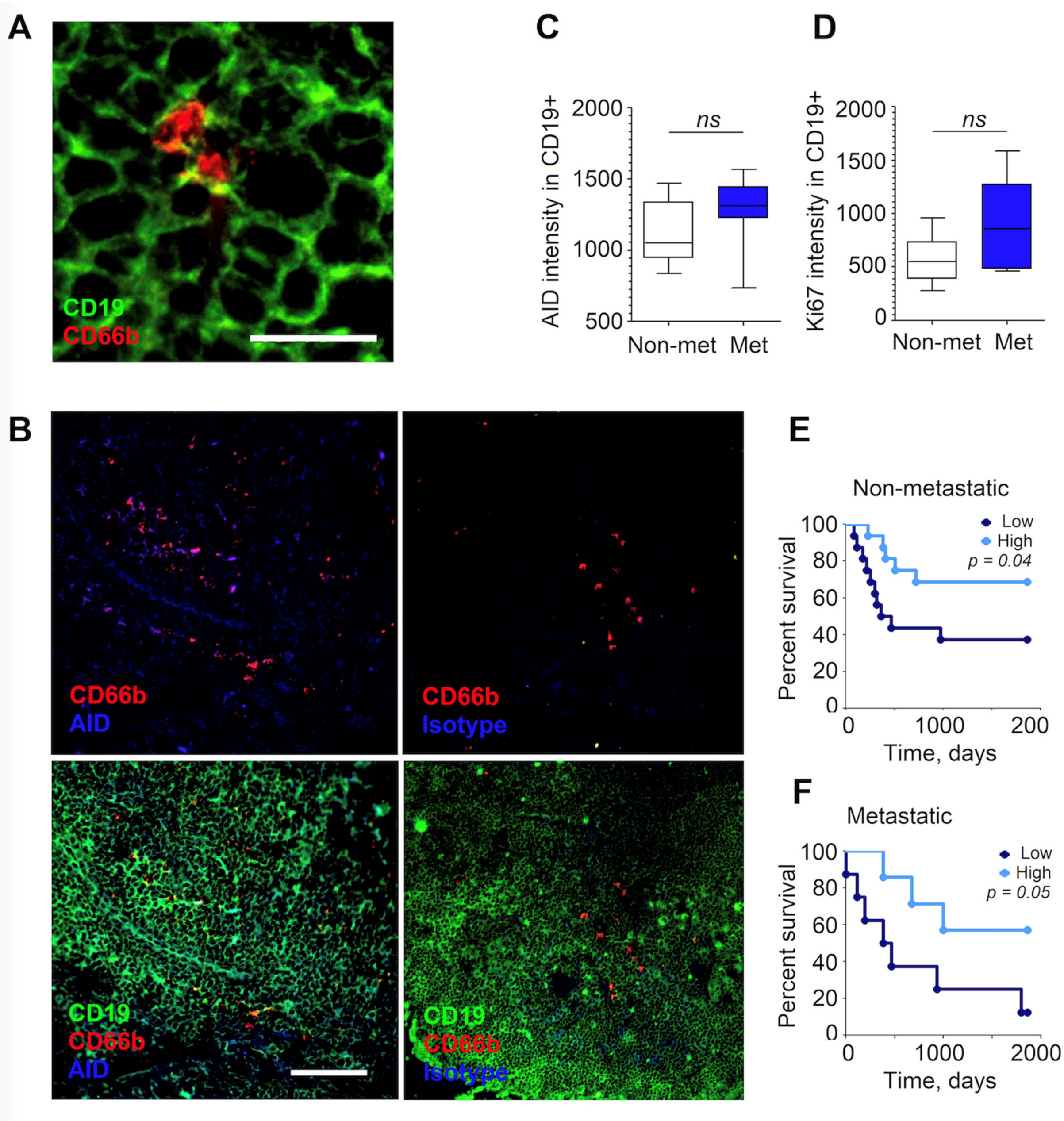

Figure S1. NBH interact with B cells in both metastatic and non-metastatic RLN (**A**) Neutrophils (red) co-localize with B cells (green) in RLNs, scale bar 12.5 µm. (**B**) Specific signal provided by AID antibody staining and the absence of a signal of isotype control, B cells (green), neutrophils (red), AID/isotype control (blue), scale bar 50 µm. (**C**) AID intensity in B cells from non-metastatic (white) and metastatic (blue) RLNs in HNC patients. (**D**) Ki67 intensity in B cells from non-metastatic (white) and metastatic (blue) RLNs in HNC patients. (**E**,**F**) Overall survival of HNC patients increased

with high (light blue) neutrophil count when compared to low (dark blue) neutrophil count, when estimated in metastasis-free (**E**) and metastatic RLNs (**F**). For nonparametrically distributed samples, a Kruskal–Wallis ANOVA with the Bonferroni correction for multiple comparisons and a Mann–Whitney U-test for two independent samples or a Wilcoxon test for dependent samples were used. Correlations were analyzed with a Spearman R test. Kaplan–Meier curves for the survival function were compared via a log-rank-test. $p < 0.05$ was considered significant.
